# Rapid and Sensitive Detection by Combining Electric Field Effects and Surface Plasmon Resonance: A Theoretical Study

**DOI:** 10.3390/mi15050653

**Published:** 2024-05-15

**Authors:** Qijie Qiu, Yan Xu

**Affiliations:** School of Mechanical Engineering, University of Xinjiang, Urumqi 830049, China; 19945844795@163.com

**Keywords:** surface plasmon resonance, dielectrophoresis, AC electroosmosis

## Abstract

Surface plasmon resonance (SPR) has been extensively employed in biological sensing, environmental detection, as well as chemical industry. Nevertheless, the performance possessed by conventional surface plasmon resonance (SPR) biosensors can be further limited by the transport of analyte molecules to the sensing surface, noteworthily when small molecules or low levels of substances are being detected. In this study, a rapid and highly sensitive SPR biosensor is introduced to enhance the ability of the target analytes’ collection by integrating AC electroosmosis (ACEO) and dielectrophoresis (DEP). Both the above-mentioned phenomena principally arise from the generation of the AC electric fields. This generation can be tailored by shaping the interdigitated electrodes (IDEs) that also serve as the SPR biomarker sensing area. The effects exerted by different parameters (e.g., the frequency and voltage of the AC electric field as well as microelectrode structures) are considered in the iSPR (interdigitated SPR) biosensor operation, and the iSPR biosensors are optimized with the sensitivity. The results of this study confirm that the iSPR can efficiently concentrate small molecules into the SPR sensing area, such that SPR reactions achieve an order of magnitude increase, and the detection time is shortened. The rapid and sensitive sensor takes on critical significance in the development of on-site diagnostics in a wide variety of human and animal health applications.

## 1. Introduction

To investigate the phenomenon of surface plasmon resonance (SPR), a spectroscopic method has been proposed, which allows for the real-time and label-free [1] monitoring of the interactions between the free analytes in a solution and a biomolecular recognition element immobilized on the surface of the sensor for detecting and capturing a target analyte of interest [2]. SPR provides the real-time and label-free measurement of kinetics and affinity of bimolecular binding. SPR confers a significant advantage over radioactive or fluorescent labeling methods since labels may impair bindings. Specifically, SPR is cost-effective, can directly measure the binding constant and affinity, and leads to less reagent consumption [3]. Accordingly, the use of SPR biosensors has become increasingly popular in fundamental biological studies, health science research, drug discovery, clinical diagnosis, and environmental and agricultural monitoring. Nevertheless, the conventional SPR signal response decays exponentially to the outside in the direction perpendicular to the interface, and it is less sensitive to the changes in RI that exceed the surface of the sensing interface by 200 nm [4]. Thus, it is difficult for SPR biosensors to detect large-scale targets (e.g., cells, bacteria, and even exosomes) [5].

It is generally known that, regarding the conventional SPR, the binding events consist of a depth of 200 nm from the thin metal layer, a distance at which the analyte–ligand interaction influentially disturbs the plasmonic wave and produces a detectable signal [6]; hence, the current of the SPR biosensors is often limited by diffusion-limited mass transport [7]. To improve the sensitivity of the SPR sensor system and conform to the needs of the ultra-sensitive detection of trace disease biomarkers, the commonly used method is to enhance the detection sensitivity and stability of SPR by introducing nanomaterials and different topology structures. Nanomaterials (e.g., graphene and molybdenum disulfide) possess a larger specific surface area, high electron mobility, and light absorption rate; a sensitization layer to enhance the SPR signal can be built by the aid of nanomaterials without disturbing the interaction of the biomolecules, which can increase the sensitivity of the SPR and provide precise detection for trace biomarkers [8]. Zhihui Mao proposed a method via graphene to change the surface of the sensor chip to detect PD-L1 exosomes, and the detection limit of 20 particles mL^−1^ was reached without signal amplification [9]. Yindian Wang developed a rapid and sensitive SPR sensor based on a two-dimensional metal–organic framework (2D MOF Cu-TCPP), and the LOD for PD-L1 exosomes was obtained as 16.7 particles mL^−1^ [10]. However, the above-described techniques require nanofabrication steps in which expensive equipment and long manufacturing processes are necessarily involved. Chung-Ting chou Chao et al. designed a tunable refractive index-based plasmonic sensor. This sensor consists of an MIM waveguide connected with a nanoring containing Ag nanorods, which have a significant impact on the structure’s sensitivity. The sensitivity of this design was 2080 nm/RIU [11]. However, these biosensors exhibit performance limited by the poor diffusion of the target molecules, exemplified by a large depletion zone near the sensor surface, especially for high-density microarrays. To conduct the current SPR sensor sensitization works, there is an urgent need to propose a method for increasing the mass transport rate and enriching the target analyte into the SPR sensor surface rapidly. This urgently required method can be ideal and highly promising in enhancing the performance of SPR sensors.

The limitations of the above-mentioned SPR sensitization methods can be addressed by increasing the analyte concentration near the sensor surface with active mass transport, e.g., by employing [12] an externally applied electric field. A novel method to increase the sensitivity is to combine DEP and ACEO to rapidly enrich the target analyte on the sensor surface [13]. DEP refers to the motion of the polarized particles in a non-uniform electric field that arises from the formation of an induced dipole because of the relative permittivity of the particle and the surrounding medium [14]. Under the effect of DEP, particles move toward the region of the greatest field intensity (e.g., an electrode), and these particles can be modified by adjusting the input AC frequency and the conductivity of the medium [15]. ACEO has been reported as an electrokinetic phenomenon that occurs when the tangential component of the electric field moves the ions in the ionic double layer at the metal/medium interface [16]. The above-mentioned techniques have been employed in the existing research for separating, collecting, and concentrating bacteria, cells, and DNA.

For instance, Euisang Yu proposed a vertical nanogap architecture that possesses an electrode–insulator–electrode stack structure, which can expedite the generation of strong DEP and ACEO under low voltages so as to precisely capture and spatiotemporally manipulate nanoparticles and molecular assemblies [17]. Avijit Barik discovered that nanohole-enhanced DEP and ACEO enabled the real-time label-free detection of analyte molecules in a 5 μL droplet in a few minutes [18].

In this study, the sensing strategy adopted a newly developed approach for the active mass transport with SPR based on interdigitated electrodes (IDE) that significantly enhances the target collection ability. iSPR sensing aims to break the fundamental diffusion-limited barrier to increase the sensitivity, reduce the detection time and LODs, and eventually enable multiplex sensing to achieve high-throughput, high-integration sensing platforms [19]. Compared to the method of the different topology structures and nanomaterials to enhance plasmonic sensors’ sensitivity and applications, iSPR sensing can selectively extract target analytes from the complex biological samples through the positive dielectrophoretic force and negative dielectrophoretic force generated between the electrode pairs. This effectively improves the specificity of the biosensors. At the same time, we provide the results of the numerical simulation and enrichment of polystyrene particles. The experimentally verified iSPR chip is easily adaptable to the existing SPR sensing systems, which are commercially available without modifying their optical setup.

## 2. Theoretical Background

This study considers the coupling effect of DEP and ACEO on the dynamic detection process of target analytes. This section is divided into subsections labeled as development of DEP–ACEO-enhanced SPR system and dielectrophoretic- and AC electro-osmotic characterization.

**Figure 1:**
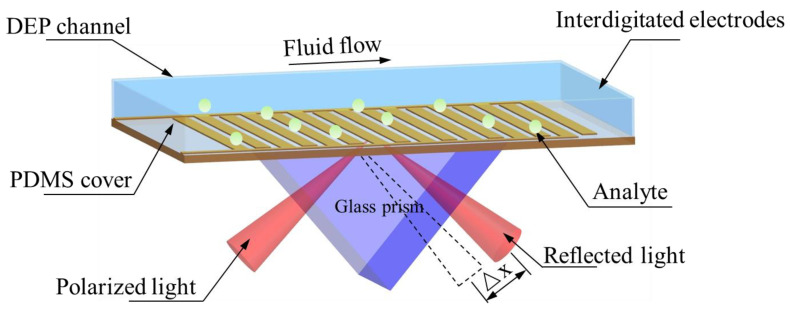
Schematic and principle of AC DEP–ACEO-enhanced surface plasmon resonance. Au microelectrodes are first patterned by photolithography and metal liftoff on a glass substrate. With out-of-phase AC voltage applied to the Au electrodes, the electrical double layer horizontally moves along the electrode surfaces. The electrical double-layer motion generates a hydrodynamic rotational flow in the microfluidic channel, and the target analyte is polarized. The hydrodynamic flow (ACEO) and DEP facilitate the transport of the target biomolecules to the sensing surface and their surface binding reaction.

Development of DEP–ACEO-enhanced SPR system. Figure 1 depicts the schematic diagram of the iSPR system. Our device is composed of a glass substrate with comb-shaped Au microelectrodes and another polydimethylsiloxane (PDMS) microfluidic channel layer mounted on the prism in the Kretschmann configuration. Specifically, the PDMS layer is attached onto the glass substrate, and the sample detection channels (100 µm in height) are orthogonal to the comb-shaped Au microelectrodes. The optical contact between the prism and the SPR biochip is generated by employing indicator matching oil. The primary optical components around the iSPR chips are composed of a light source, a prism coupler, as well as a detector (camera).

The surface of interdigitated electrodes serves as the SPR sensing area. Under the electric field, a rotational fluid motion is generated by the motion of ion charges in the electrical double layer, which are induced on the electrode surfaces. The above-mentioned fluid motion results in microfluidic agitation, which can contribute to the target transport of analytes down to the electrode surfaces. Since the surface of the electrode acts as the effective area of the DEP force, target analytes in solution bind to the ligands on the surface of interdigitated electrodes in the presence of a strong electric field gradient, which can adjust the incident light refractive indicator near the chip surface. In other words, increasing the number of analyte molecules bound to the sensor surface can lead to the enhancement of the sensor signal.

Dielectrophoretic and AC electroosmotic characterization in iSPR chips. By using a non-uniform electric field, target analytes are sufficiently protected from the dielectrophoretic (DEP) force. DEP exerts attractive or repulsive forces directly on particles, which is dependent on AC signal frequencies as well as (di)electrical properties of particles and the sample solution. In accordance with the principle of dielectrophoretic force, the time-averaged DEP force experienced by a homogeneous spherical particle with radius r in a medium of permittivity $\varepsilon_{m}$ is written in the following function form [20]:

(1) FDEP =2πεmr3Re[fCM]∇|E|2

The polarity (positive or negative) of the DEP force is ruled by the dielectric properties of cells relative to the surrounding medium, where Re[f_CM_] represents the real part of Clausius–Mossotti factor, which satisfies

(2) $$f_{\mathrm{CM}}=\frac{\varepsilon_{p}^{*}-\varepsilon_{m}^{*}}{\varepsilon_{p}^{*}+2\varepsilon_{m}^{*}}$$

where $\varepsilon_{p}^{*}\;$and $\varepsilon_{m}^{*}$ express the complex dielectric properties of the particle and surrounding medium, respectively, $\varepsilon^{*}=\varepsilon -j\sigma /\omega \;$ (σ and ω are conductivity and angular frequency, respectively). If analytes are more polarizable than the suspending medium, Re[f_CM_] becomes positive, and analytes move towards the high field region around the electrodes under the effect of the positive DEP force [21]. In contrast, if Re[f_CM_] is negative, target analytes will be pushed away from the high-electric field region by a negative DEP (nDEP) force. Compared with ACEO, the DEP force possesses the capability of selectively trapping and expelling suspended target analytes from the interdigitated electrodes where the maximum $\nabla |E{|}^{2}$ gradient is generated, which increases the local concentration of the target analytes in SPR reactions. 

We yield the maximum $\nabla |E{|}^{2}$ as

{(3)∂E(x,y)2∂x=2ExExx+2EyEyx(4)∂E(x,y)2∂y=2EyEyy+2ExExy

Concomitant to ACEO effects, for spherical analytes, the DEP velocity is defined by Equation (5) [22] as follows:

(5) uDEP =r2εm6ηRe [εp*−εm*εp*+2εm*]∇|E|2

The moving ions and charges form a directional fluidic motion near the surface, which is termed a.c. electroosmosis flow [23]. The electrokinetic phenomenon is capable of generating strong three-dimensional (3D) vortices under relatively low voltages, improving mass transfer-enhanced analyte–receptor interactions, and enhancing biosensing performance. 

The velocity v of the flow induced by the ACEO at the frequency f near the microelectrode surface satisfies

(6) $$u_{ACEO}=\frac{\varepsilon \varphi^{2}\Omega^{2}}{8\mu x{\left(1+\Omega^{2}\right)}^{2}}$$

where φ, μ, and η denote the peak value of the applied potential, permittivity, and dynamic viscosity of the fluid, respectively, and the non-dimensional frequency of Ω is formulated in the following function form:

(7) $$\Omega =\frac{1}{2}\pi \kappa x\left(\frac{\varepsilon_{m}}{\sigma_{m}}\right)\omega$$

where x denotes cross-sectional position and starts from the center of the gap between the microelectrodes, and k represents the reciprocal of the Debye length. The above equation is generic and may apply to electrodes of varying geometries. Notably, only the values of the function that falls into the range of the electrode are adopted. The function cannot apply to regions above the electrode gap where there is no induced counter ion [24]. During iSPR detection, while pDEP typically attracts objects to the edge of electrodes, ACEO is capable of overcoming strong DEP effects and dragging them toward the center of the electrodes, which can be conducive to SPR detection.

Specifically, the concentration change and diffusion velocity of the target analyte in the iSPR chip were calculated, respectively, and the force of the particle was analyzed so as to accurately predict the enhancement of the DEP–ACEO target analyte transport and the increased binding efficiency of analytes to probes immobilized on the electrode surface. Compared with conventional SPR, the effectiveness of the proposed method in this study in increasing detection efficiency is validated. The ACEO velocity serves as the slip boundary condition in the concentration field model under the action of the AC electric field. Subsequently, the transient local analyte concentration is computed by solving the mass transport equation that accounts for diffusion and convection, which is provided by

(8) $$\partial_{c}/\partial_{t}+(u_{DEP}+u_{ACEO})\nabla c=D\nabla^{2}c$$

If there is no external disturbance in low-Reynolds-number fluids, the fluid always maintains laminar flow, and the mass transfer process is dominated by diffusion. The concentration c satisfies

(9) $$\partial_{c}/\partial_{t}=D\nabla^{2}c$$

To evaluate the transient dynamics of a spherical analyte (with its mass of m_p_) in iSPR biosensor chips, the Langevin equation of particle velocity (**u**_p_) is employed [25], which is expressed as

(10) $$m_{p}\frac{{du}_{p}}{dt}\;\;=F_{\mathrm{DEP}}+F_{\mathrm{ACEO}}+F_{\mathrm{grav}}+F_{\mathrm{buoy}}+F_{\mathrm{int}}+\xi \left(t\right)$$

Based on DEP force (**F**_DEP_), Stokes drag force induced by ACEO flow (**F**_ACEO_), gravitational force (**F**_grav_), buoyant force (**F**_buoy_), interparticle force from Coulomb interaction (**F**_int_), and random Brownian force (ξ(t)), Stokes drag forces are defined as

(11) $$F_{\mathrm{ACEO}}=-6\pi \eta r(u_{p}-u_{m})$$

where **u**_p_ and **u**_m_ represent the velocities of target analytes and fluidic flows, respectively, and **F**_ACEO_ acting on a single particle can be evaluated by importing **u**_ACEO_ into **u**_m_. Since the iSPR chips possess low concentrations, **F**_DEP_ and **F**_ACEO_ principally determine the motion trajectory and direction of target analytes, whereas the others are negligible. In accordance with the above-mentioned theory, when the dielectrophoretic force from the AC electric field exceeds the drag force from the flowing fluid, the target analytes can be captured in the AC electric field rather than flowing with the fluid for in situ biomarker detection.

Since the typical experimental observation time (t) is pronouncedly longer than the relaxation time (τ) of the target analytes, the relaxation time (τ) satisfies Equation (12):

(12) τ=m/6πηr

Since the relaxation time (τ $\approx 1.47\times 10^{-6}\;s$) is significantly shorter than the experimental observation time, the transfer speed of target analytes in the iSPR biosensor chips is expressed as

(13) $$u_{p}={(u}_{ACEO}+\frac{F_{DEP}}{12\pi \eta r})\left(1-e^{-t/\tau }\right)\approx \;u_{ACEO}+u_{DEP}$$

The initial velocity of the target analytes is assumed to be 0. Thus, the motion trajectory and direction of the suspended analytes under different AC conditions can be evaluated by calculating two dominant electrokinetics of DEP and ACEO.

In the absence of an AC electric field, the target analytes of the solution can reach the identification element surface by laminar flow as the solution sample flows through the SPR detect area. The diffusion mass transfer rate of analytes to be detected from the answer to the sensor surface can satisfy

(14) $$V_{s}\;=\frac{2}{9}\frac{r^{2}(∆\rho )g}{\eta }$$

Another performance indicator for the evaluation of iSPR biosensor chips is selectivity, which indicates whether the sensor can pick up a certain analyte. Notably, selectivity is avoided in this study [26] since it is connected to the experimental outcomes.

## 3. Results and Discussion

In the present section, the results of the proposed DEP–ACEO-enhanced SPR system on the detection performance are discussed. Prior to the discussion of the results, the theory of the iSPR biosensor chip design and its construction are first analyzed.

### 3.1. Numerical Simulations for iSPR Biosensor Chip Design

The iSPR chips employed in this study are composed of a coplanar interdigitated Au electrode pattern fabricated on a glass substrate and a microfluidic channel cut out from a 100 μm thick PDMS film obtained through spin coating. Four microelectrode patterns were developed with a fixed electrode width (E_W_) of 100 μm and electrode gaps (E_G_) of 5, 10, 15, and 20 μm. The coplanar interdigitated geometry enables the manipulation of target analytes to overcome the fluidic drag force and drive the analyte molecule to adsorb on the SPR sensor surface. DEP acts as the predominant force responsible for this phenomenon.

**Figure 2:**
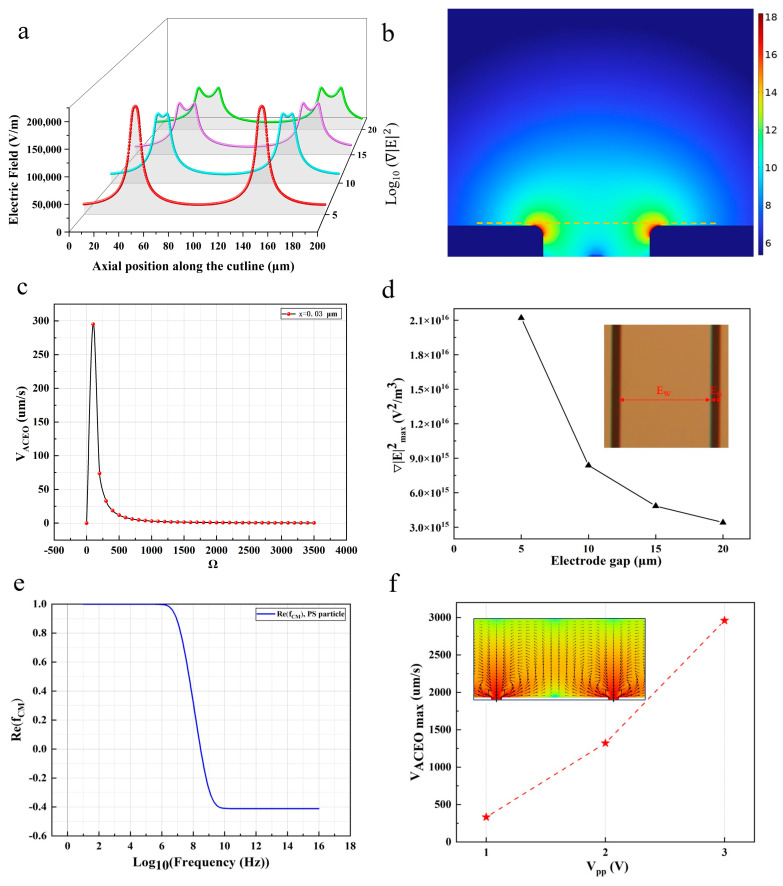
(**a**) Numerical simulation of the electric field strength under different electrode gaps. (**d**) $\nabla |E{|}^{2}$_max_ calculated under different electrode gaps as a linear function of Vpp. (**c**) Relationship between V_ACEO_ and frequency. The AC electroosmotic velocity calculated at locations ‘x’ from the electrode edge under 0.0002 S m^−1^, 1 V. (**e**) Clausius–Mossotti factor calculated for polystyrene particles ($\varepsilon_{p}=10,\sigma_{p}=1S/m$) in DI water. (**b**) Calculated distribution of $\nabla |E{|}^{2}$ with a gap distance of E_G_ = 10 μm and f = 10^5^ Hz. (**f**) The maximum value of ACEO as a result of increasing V_pp_ (f = 10^5^ Hz) from 0 to 3 V.

Based on Equations (1) and (2), F_DEP_ is computed by physical factors (e.g., the particle size, frequency-dependent electrical properties of the particles and media, Re[f_CM_], and a gradient of the E-field squared). Unlike the other factors, $\nabla |E{|}^{2}$ is derived from the structural characteristics of the microelectrodes. To select the optimal structure of the microelectrodes, the electric field, the $\nabla |E{|}^{2}$ distribution (Figure 2a,b), is calculated based on different electrode configurations with the COMSOL Multiphysics 6.1. 

In accordance with the simulation results, $\nabla |E{|}^{2}$ is indicated to be the strongest at the microelectrode (Figure 2b) and weakens farther away from the edges, which is similar to the electric field distribution (Figure 2a). Figure 2a depicts the magnitude of the electric field plotted along d = 2 µm. **F**_DEP_ attracts analyte molecules toward the edge of the microelectrodes since the E-field squared possesses the strongest gradient (red color) along the rim of the electrodes. For the improved enrichment of target analytes, the generation of higher values of $\nabla |E{|}^{2}\;$is beneficial for the dielectrophoretic properties in the capture of target analytes. By extracting and plotting the maximum value of $\nabla |E{|}^{2}\;({\nabla |E|}_{max}^{2}$) as a function of the electrode gap (Figure 2d), the linear relation between the above-described two parameters can be expressed as the increase in the electrode gap and the decrease in $\nabla |E{|}^{2}$. The above result reveals the enhanced DEP performance of iSPR in the target analytes’ capturing irrespective of the gap distance, and the $\nabla |E{|}^{2}$ reaches its maximum (${\nabla |E|}_{max}^{2}=$2.12 × 10^16^ V^2^/m^3^) at V_pp_ = 1 V and f = 10^7^ Hz. However, given the difficulty of iSPR chip processing, we select an electrode gap of 10 µm to keep our device fabrication practically accessible. In the absence of the forced convection of the sample fluid, a smaller electrode gap provides greater **F**_DEP_ and pronouncedly increases the increase in terms of the order of magnitude regarding the SPR response.

### 3.2. iSPR Biosensor Chip Fabrication

The microelectrodes used were fabricated through photolithography on silicon wafers. The glass chips purchased from Taobao Market were cleaned with a piranha solution (H_2_SO_4_:H_2_O_2_ = 3:1) for 10 min and washed three times with pure water. Moreover, the glass substrate was coated with a 5 nm chromium adhesion layer and a 48 nm gold layer on top. The AZ1512 positive photoresist was spin-coated on the sample and post-baked at 110 °C for 3 min. Then, the photoresist was exposed to ultraviolet light through a mask superposed on the sample before being patterned by the MF319 developer (Shipley). The Au layer was then patterned by wet etching with a KI3 solution to remove the exposed gold film. Subsequently, the Cr layer was removed by using a Cr etchant. The AZ1512 pattern on the glass substrate was removed using acetone, and then ethanol and deionized water were used to clean the chips.

### 3.3. Tuning of Critical Elements in DEP and ACEO

We further varied the applied frequency and voltage with the microelectrode structures fixed at E_W_ = 100 µm and E_G_ = 10 µm. We evaluated how the AC frequencies affect the motion of the suspended target analytes through two dominant factors of the CM factor and $u_{ACEO}$. The frequency of the AC electric field not only affects the direction of the target analytes’ motion but significantly affects the speed of the analytes in the solution system. We classified the frequency ranges into three regimes: low frequency (1–10 kHz), mid frequency (0.1–1 MHz), and high frequency (>10 MHz), where each regime is expected to be principally affected by positive DEP (pDEP), ACEO, and negative DEP (nDEP), respectively.

The direction of **F**_DEP_ can be controlled by adjusting the frequency because **F**_DEP_ highly depends on the polarizing behavior of particles. Figure 2e represents the CM factor for 5 µm latex beads in DI water and is calculated with MATLAB R2022a. For instance, the particles move along the increasing field gradient at the condition of pDEP, where Re[f_CM_] > 0, whereas the repulsion of the particles from the region of the highest field gradient is referred to as nDEP, where Re[f_CM_] < 0 [27]. While positive DEP trapping occurs over a wide range of frequencies, the DEP force decreases rapidly as we move away from the interdigitated electrodes. Thus, the DEP force alone may not be effective in trapping the molecules from the bulk solution because of the diffusion-limited transport. In our experiments, a low-conductivity solution minimized any electrothermal flows, but we expected electroosmotic flows.

Since $u_{ACEO}$ scales with f (Equations (6) and (7)), our simulation in Figure 2c shows the AC electroosmotic velocity as a function of the frequency and position on an electrode. The AC electroosmotic velocity is always the highest at the edge. It decreases as the center approaches, which can act in concert with DEP to facilitate the transport and subsequent capture of the analyte molecules on the plasmonic hotspots of the interdigitated electrodes. As depicted in the above figure, the AC electroosmotic velocity is almost zero at low and high frequencies and reaches a maximum at mid-range frequencies. A higher AC electroosmotic flow rate can effectively improve the mass transfer-enhanced analyte–receptor interactions and shorten the SPR detection time, thus enhancing the biosensing performance. Considering the influence factors of DEP and ACEO comprehensively, the best operating frequency of f = 10^5^ Hz for the optimal detection frequency is determined.

To explore the practical utility of the iSPR device for clinical diagnosis, we further achieved high ACEO velocity by adjusting the magnitude of the AC voltage. As the applied V_pp_ increased from 1 V to 3 V at a fixed frequency of 10^5^ Hz, the ACEO velocity gradually increased (Figure 2f), and the analyte–receptor interactions were enhanced with the voltage amplitude. The velocity of the electroosmotic flow reached its maximum ($u_{ACEO}=2960$ μm/s) at V_pp_ = 3 V. The above-described low-voltage operation acts as one of the critical prerequisites for the practical application of iSPR biosensor chips for clinical diagnosis since it eliminates any possible scenarios of undesirable molecular phase transitions or thermal denaturation. The above-mentioned additional effects adversely affect the stability of the iSPR chips’ sensor performance and are difficult to predict theoretically.

### 3.4. Forces Acting on Target Analytes’ Dynamics under AC Electric Field

After the optimal iSPR biosensor chips’ operation condition is determined to be V_pp_ = 3 V at f = 10^5^ Hz through the above theoretical analysis, using the mechanical model of target analytes’ diffusion transport under the electrokinetic effects, how the AC electric field affects the motion of the suspended target analytes is evaluated. The simulation results suggest that the **F**_DEP_ and **F**_ACEO_ dominantly determine the motion of the polystyrene (PS) particles. Moreover, the other influencing factors are negligible in the low-concentration solution.

**Figure 3:**
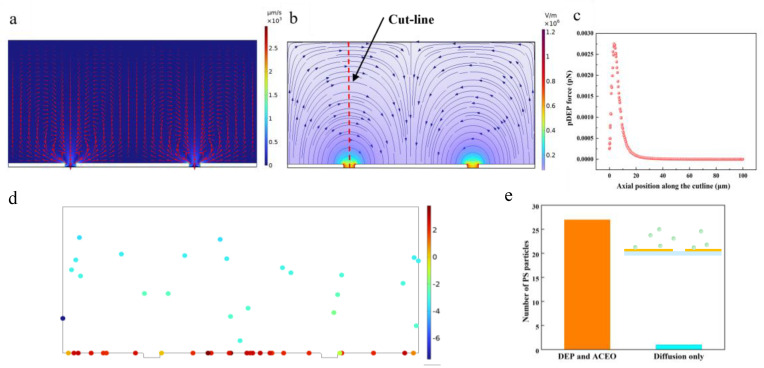
Simulation of the electric fields for the evaluation of DEP and ACEO. (**a**) Calculated distribution of ACEO flow on electrode surfaces under V_pp_ = 3 V and f = 10^5^ Hz. (**b**) Numerical simulation of the electric field strength in iSPR biochips. (**c**) Details of the DEP force at distances 5 μm from the electrode edge along the red dashed line in (**b**). (**d**) Plot of log10(${|F}_{DEP}|/{|F}_{drag}|$) comparing the forces in vertical direction exerted on PS particles under AC electric field (dielectrophoretic vs. Stokes drag force). (**e**) The number of captured PS particles in the SPR sensing area at 15 s.

Calculating **F**_ACEO_. The incompressible Navier–Stokes simulation is subjected to the electroosmotic flow. The boundary conditions for this simulation include no slip on the channel walls and zero pressure at the inlet and outlet. The boundary condition of the microelectrodes refers to a slip condition determined through the ACEO analysis, and the AC electroosmosis velocity is covered in the slip boundary condition to determine the velocity field of the solution. The above-mentioned method was adopted on the surface of each electrode with Equation (6). Since the equation aims to be centered on a microelectrode gap, it may apply to a pair of microelectrodes. However, since three microelectrodes are employed in this model, Equation (6) should be used twice—once centered on the left microelectrode gap and once centered on the right microelectrode gap. Based on the theoretical calculation above, ACEO played a significant role in the drag force when the amplitude was V_pp_ = 3 V and the frequency was 10^5^ Hz, which can be verified by the flow fields shown in Figure 3a.

Calculating **F**_DEP_. We expected to use the pDEP force to construct traps that are adopted to hold micron-size beads at selected locations on interdigitated electrodes. To investigate the trapping force acting on the polystyrene particles [28], 5 μm diameter PS particles were adopted to simulate the phenomena by using the physical values of V_pp_ = 3 V and $\varepsilon_{m}$ = 80·ε_0_. Our quantitative models of the pDEP suggest that the microelectrodes hold maximum forces for polystyrene particles in the iSPR biosensor chip of ~1 pN (at 3 V_pp_). To further clarify the enrichment effect of DEP on the target analytes, we considered the magnitude of the y-directional DEP force along the red dashed line in Figure 3b. As revealed by the result, **F**_DEP_ reaches its maximum at the edge of the microelectrodes in Figure 3c, where the analytes accelerate towards the edge of the microelectrodes and the enhancement of the SPR signal.

A comparison between these two effects on the PS particles’ transport is shown in Figure 3d, which is the ratio of the norms of **F**_DEP_ vs. the Stokes force (**F**_ACEO_). The ratio plot shows that the DEP function as a short-range trapping force for the polarizable particles is stronger (ratio > 1.0) near the microelectrodes’ surface (within ∼5 µm). At the same time, we also analyzed the enrichment quantity of the PS particles in the SPR detection area within 15 s through the numerical calculation in Figure 3e. The results showed that the enrichment number of the PS particles under the influence of the two physical mechanisms of DEP and ACEO is much higher than that of the PS beads under the static incubation driven by pure diffusion. From the above analysis, we know that, compared with conventional SPR, iSPR biosensor chips increase the detection range and can increase the enrichment amount of the target analytes in a shorter time, thus shortening the detection time.

### 3.5. Target Analytes’ Collection Using the iSPR Biosensor Chip

**Figure 4:**
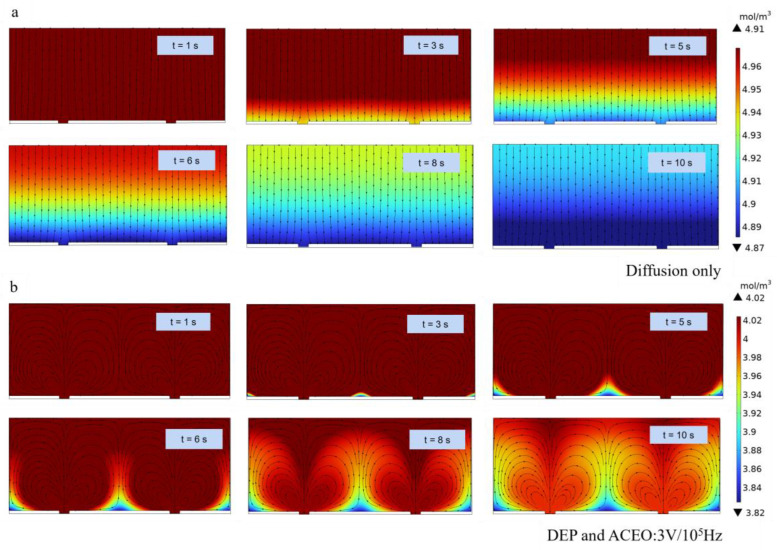
Target analyte concentration profiles in the iSPR microfluidic channel of the device operated under DEP and ACEO at V_pp_ = 3 V f = 10^5^ Hz, and with diffusion only, respectively. The flow field direction is shown by the black arrows. The initial concentration for two cases is set at 5 mol/L. The inlet (left edges) and the outlet (right edges) for diffusion-only cases (**a**) and DEP–ACEO (**b**) are defined as open boundaries with no target analyte replenishment.

We compute the target analytes’ concentration at the initial time (t = 0 s) and after an incubation period of 10 s for two assay conditions through numerical experiments: (1) DEP and ACEO-agitated incubation at V_pp_ = 3 V and f = 10^5^ Hz (Figure 4b), and (2) static incubation driven by pure diffusion without DEP and ACEO (Figure 4a). The initial value of the target analytes’ concentration at the inlet of the microchannel is derived as 5 mol/m^3^.

Compared with the case in Figure 4b, for the distribution of the target analytes’ concentration without the DEP and ACEO effect, the flow was wavy, and the replenishment of the consumed analyte close to the SPR sensing area was affected only by the convection–diffusion. Accordingly, the binding reaction between the target analytes and the immobilized ligand was restrained by the viscous effects near the surface reaction and limited the immunoassay performance of the iSPR biosensors. 

However, when the electric field is turned on, the analyte concentration distribution is distorted by the electroosmotic flow and DEP, and the original laminar flow distribution is broken. Thus, the ACEO effect produced a circular flow that caused fluid mixing and rapidly brought the target analytes to the binding surface. As depicted in Figure 4b, the circular flow generated by ACEO significantly accelerated the mass transfer rate of the antigen in the solution system compared with the static incubation condition in which the sample solution is not replenished at the sensor surface. Under the combination of the DEP and ACEO effects, the antigen concentration reduced near the electrode surface due to the interaction with the antibodies on the surface [29]. Thus, the electrokinetic effect enhanced the transport of the analytes toward the sensor surface, increasing the chance of the association between the antigen and antibody. 

**Figure 5:**
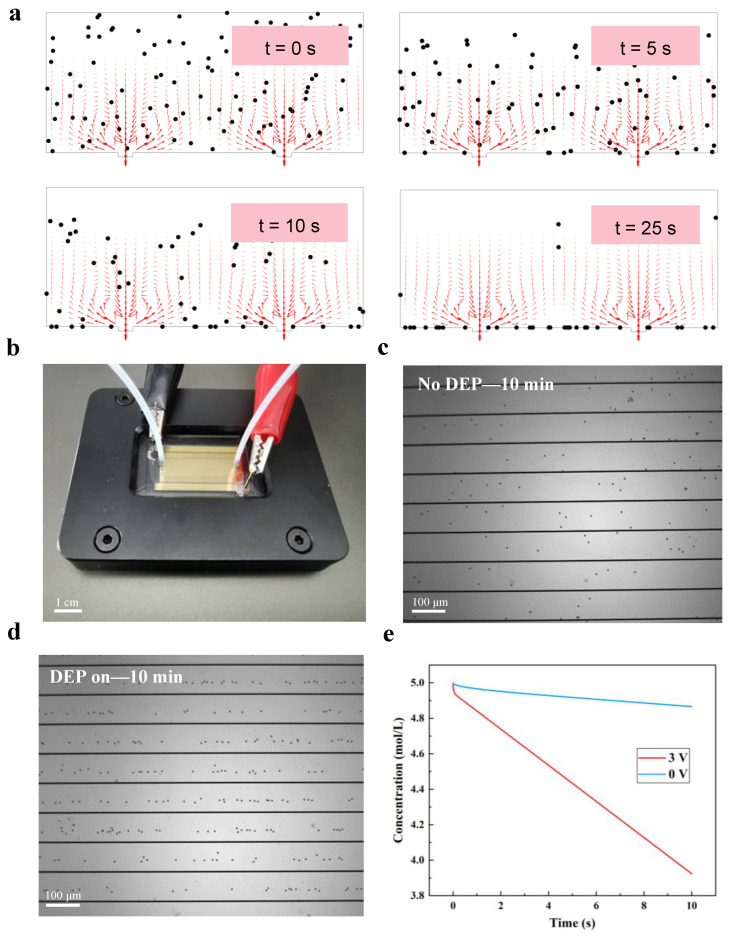
Simulation (**a**) and experimental (**b**–**e**) results of the pDEP and ACEO effects of polystyrene microbeads. (**a**) Results of the COMSOL particle tracing temporal study with a 3 V_pp_ voltage and the same electrical parameter as previously. A relative buffer permittivity of 80 is used in each model. While positive pDEP typically attracts objects to the edge of electrodes, electro-osmosis can drag microbeads toward the center of the electrodes, which facilitates SPR detection. (**b**–**d**) Representative images of polystyrene microbead collection using the iSPR chip (under 10× magnification). ISPR chip with 5 μm diameter polystyrene microbeads at 10^5^ Hz and 3 V suspended in 0.0002 S m^−1^ media with a low concentration of particles. (**e**) Comparison of the concentration changes for DEP–ACEO and diffusion only from the COMSOL temporal study.

Lastly, to evaluate the effects exerted by DEP and ACEO on the response time of SPR, the temporal evolution of the association and dissociation of the analyte–ligand complex at the SPR sensing area was numerically and experimentally analyzed with PS particles as the analysis object. Figure 5a depicts the motion of the PS particles at 0 s, 5 s, 10 s, and 25 s, respectively. The simulated results under the optimal frequency and V_pp_ indicate that the particles are subjected to strong DEP and ACEO effects. They are rapidly dragged toward the center of each microelectrode, enhancing the analyte–ligand binding. At t = 10 s, a considerable amount of the PS particles are enriched to the microelectrode’s surface, meaning that the complex concentration on the SPR sensing area increased steeply over a short time.

Next, we experimentally demonstrated the detection of low-concentration PS particles using the iSPR biosensor chip to clearly demonstrate the effects of the DEP and ACEO fluid mixing operation on the SPR response time. To this end, we computed the particles’ natural diffusion mass transfer rate as 0.68 μm/s with Equation (14) and the average velocity of the particles under the effects of the DEP (5.05 μm/s) and ACEO (1480 μm/s) with Equations (5) and (6). Notably, the promoting effect of the AC electric field on the detection time of SPR was obtained via formula calculation.

To monitor the real-time enrichment of the PS particles on the sensor surface, we first conjugated and fixed the iSPR biosensor chip with a clamp as in Figure 5b and slowly injected PS buffers into the biosensor chip. Furthermore, we recorded the particle distribution change in the SPR sensor pattern at 10 min by fluorescence microscopy and compared it to that without DEP and ACEO (in Figure 5c,d). When several particles are collected, the particles tend to align in rows [30] called pearl chains, as the model predicts. As a result, the capture efficiency of particles is very low without DEP and ACEO, as in Figure 5c. Then, we conducted a kinetics analysis of the changes in the target analytes’ concentration over time to calculate a decline rate through the theoretical model for the DEP and ACEO-enhanced analyte transport and surface reaction in Figure 5e. As depicted in Figure 5, with the association of the analyte–ligand complex at the electrode surfaces, the concentration of the target analytes in the microchannel gradually decreased. Furthermore, DEP and ACEO accelerated the binding reaction.

## 4. Conclusions

In this study, we have uniquely integrated the DEP and ACEO effects with surface plasmon resonance biosensors. Through the 2D numerical simulation and experiment method, we evaluated the application of the combination of the DEP and ACEO effects in SPR sensing by adjusting the amplitude and the frequency. Based on the results, we have demonstrated that the adequate electrohydrodynamic agitation and kinetic adsorption in the bulk solution can significantly improve the label-free sensing performance of the surface plasmon resonance biosensors for detecting small molecules at low species concentrations. The iSPR chip allows us to use a standard Kretschmann-type SPR measurement system without an imaging function. Moreover, our device has successfully achieved high sensitivity and accuracy regarding detection.

Our future development of an automated and faster detection system for exosomes under low-voltage working conditions would enable our iSPR biosensor chips to be integrated into the system, which would put forward new requirements for the structural optimization design of iSPR biosensor chips. Therefore, we will use the catenary function modeling method further to optimize the structure of the iSPR biosensor chips. Catenary functions are pivotal in describing the electromagnetic vectors, intensity distribution, and dispersion of structured light on the subwavelength scale [31]. This numerical analysis method may help to guide the structural optimization of iSPR biosensor chips.

## Data Availability

All data that support the findings of this study are included within the article.
